# Analysis of biofilm and bacterial communities in the towel environment with daily use

**DOI:** 10.1038/s41598-023-34501-4

**Published:** 2023-05-10

**Authors:** Haruro Kato, Nagisa Okino, Hiroki Kijitori, Yoshifumi Izawa, Yasunao Wada, Masataka Maki, Takako Yamamoto, Takehisa Yano

**Affiliations:** 1 Safety Science Research Laboratories, Kao Corporation, 2606 Ichikai, Haga, Tochigi 321-3497 Japan; 2 Household Products Research Laboratories, Kao Corporation, 1334 Minato, Wakayama, Wakayama 640-8580 Japan; 3 Biological Science Research Laboratory, Kao Corporation, 1334 Minato, Wakayama, Wakayama 640-8580 Japan; 4 Intellectual Property Organization, Kao Corporation, 1334 Minato, Wakayama, Wakayama 640-8580 Japan

**Keywords:** Biofilms, Microbiology, Bacteria, Metagenomics

## Abstract

Towels differ remarkably from other textile products in their fibre structure and usage, and microbial behaviours on towels remain underexplored. Thus, we evaluated biofilm formation on towels during use for 6 months in daily life and analysed its relationship with odour, dullness, and laundry habits. The towels exhibited odour and dullness after 2 months of use and biofilm structures were observed over the 6 months, especially in the ground warp part. Polysaccharides, proteins, nucleic acids, and viable counts on the towels increased over time. The microbiota was significantly different from that on human skin and clothing*.* Several species of Alphaproteobacteria were correlated with dullness intensity and the quantity of biofilm components. Therefore, bacterial species that specifically adapt to the towel fibre environment could form biofilms. Our results demonstrate bacterial diversity in textile products and suggest careful consideration of the textile fibre material, structure, and usage pattern to control bacterial communities.

## Introduction

Various microorganisms are reported to adhere, grow, and secrete an extracellular matrix to form biofilms in environments such as textile product surfaces and gaps^1^. Although textile products are regularly washed for hygiene maintenance, washing has been proven insufficient in the removal of biofilm-forming microorganisms^2,3^. Skin microorganisms have been shown to be closely related to the microorganisms in fibres of clothing^4^. For example, individual differences, including age and sex^5^, have a greater effect on bacterial diversity on T-shirts than on the type of fabric or washing^6^. Thus, most microorganisms are thought to adhere to garment fibres with human use; however, some could move between fibres and transfer from one garment to another while washing^4,7^.

Microorganisms in textile environments cause unpleasant odours and dullness^8,9^. For example, bacteria on clothing metabolise sweat, sebum, or other precursors into volatile substances with various odours^10–12^. Further, discolouration of textile fibres can occur via the release of by-products of microbial metabolism, specifically in the form of a dye or pigmented substance excreted directly by microorganisms. Indeed, bacterial inoculation and subsequent culture formation on textiles have been reported to lead to discolouration^9,13^.

Towels must remain hygienic because they touch every body part, including the face and hands, after washing. However, unlike other textile products such as clothes, towels are not in constant contact with body parts. Towels typically comprise a bulky pile structure with a flat ground warp structure^14^. These characteristics lead us to assume that the mechanism underlying unpleasant odours and dullness that occur in towels would be different from those of other textile products. For example, the transfer of microorganisms among textile products through wash water may have a significant influence on the microbial composition of towels. However, to our knowledge, there are no reports of biofilms or microorganisms in the towel environment with daily use.

In this study, we investigated the relationship among microbiota in towels, biofilm formation, and related consumer issues when towels are used in daily life by performing a 6-month longitudinal test. Through microscopic analysis, biofilms were observed, especially in the ground warp part of the towel, which remains hidden below the pile warp on the surface of the towels. Thus, we elucidated the relationship between microbial composition and towel environment, followed by the development of a DNA extraction method suitable for biofilms that exist in deep places in towels. To the best of our knowledge, this study is the first to describe the unique biofilm structure in towels and demonstrates the diversity of biofilm structures and microbial composition in textiles with daily use. We believe that our results will provide important knowledge for maintaining towel cleanliness, which is extremely important for daily hygiene.

## Results

### Research design

**Figure 1:**
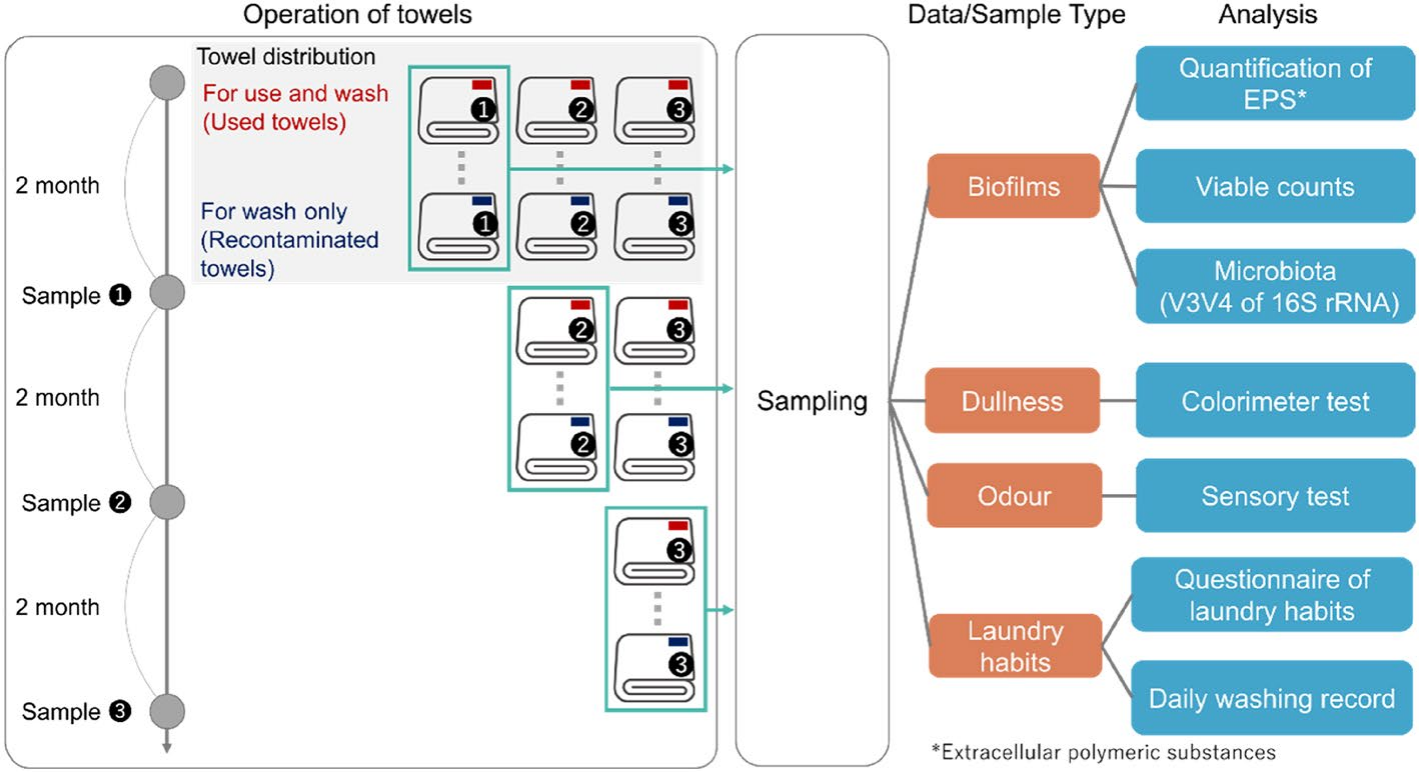
Experimental design. Six towels were prepared for each household. Three towels each were prepared with red and blue tags. Each towel was labelled from 1 to 3. Red-tagged towels were used in daily life and washed repeatedly, whereas blue-tagged towels were not used but were washed with red-tagged towels of the same number to evaluate the transfer of grime and microbes while washing. Towels were collected every 2 months and used for various analyses (orange boxes) with methods shown in blue boxes.

A 6-month longitudinal test was conducted at 26 households to analyse the odour and dullness of towels and their relationship with biofilm formation. To determine whether soils and microorganisms are transferred while washing, six new cotton towels were prepared for each household. Three were regularly used and washed (used towels), and the remaining three were not used but washed with the corresponding used towels (recontaminated towels). One of the used towels and one of the recontaminated towels with the same number of washes were collected every 2 months during the study period. A 12-cm square of the most likely area to get dirty (Supplementary Fig. 1) was cut for subsequent analyses. The survey design is shown in Fig. 1.

### Dullness and biofilm components of towels

**Figure 2:**
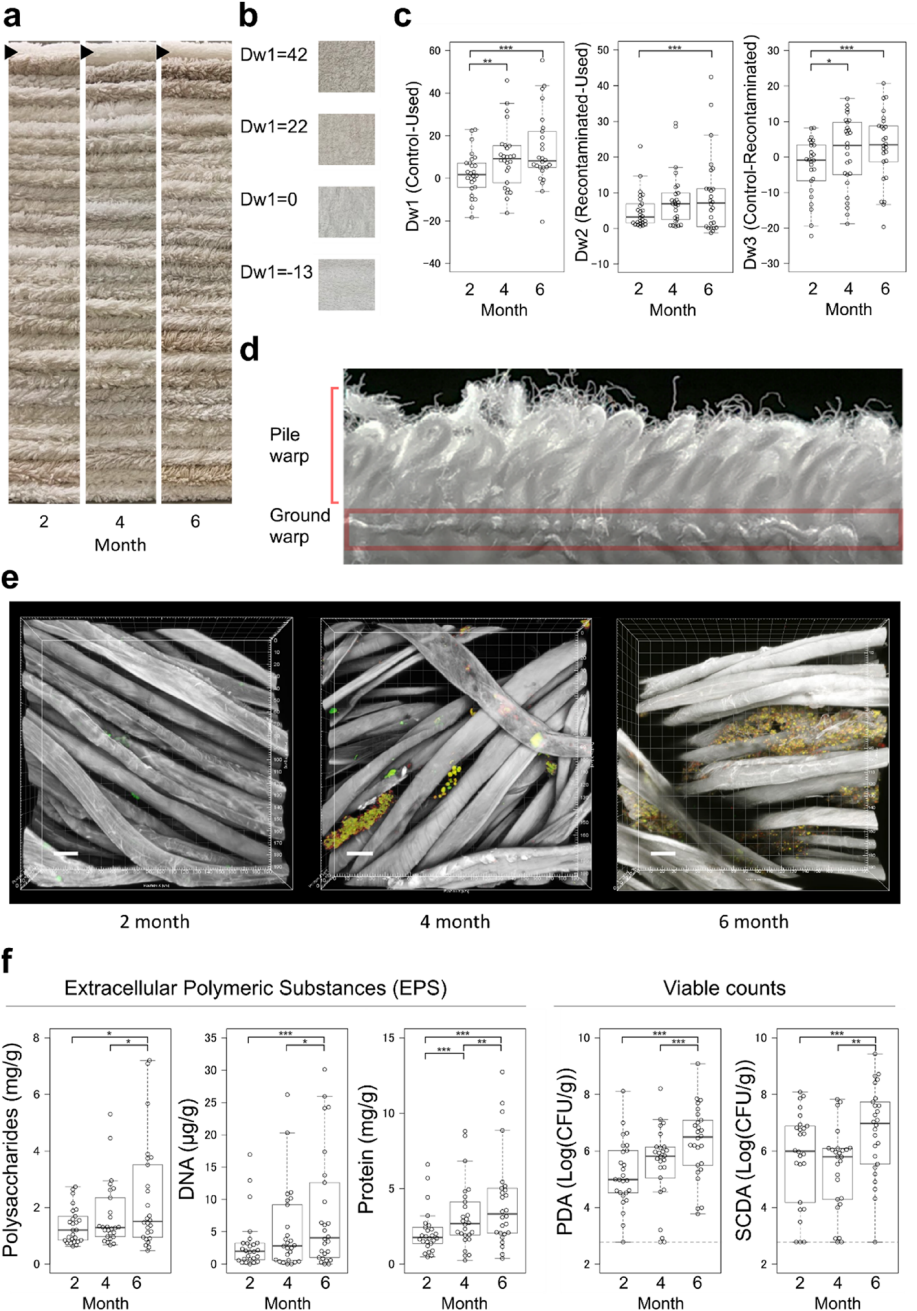
Changes in dullness and biofilm components of the used towels. (**a**) Images of the collected towels that were used for 2, 4, and 6 months. The towels were stacked and arranged by collected months. Arrowheads indicate towels that were not used or washed. (**b**) Various Dw1 values (differences in whiteness between new and used towels) and the corresponding towel images. (**c**) Difference in whiteness compared using various scales. Dw2; differences in whiteness between washed-only (recontaminated) and used-washed (used) towels. Dw3; differences in whiteness between new (control) and washed-only (recontaminated) towels. Larger Dw values indicate increased dullness. (**d**) Image of the cut surface of the towel was captured from the side using a digital camera. The pile warp covers the ground warp from both sides. The image shows the pile and ground warps on one side. The red square shows the area where biofilm formed. (**e**) Confocal microscope images of collected towels from household D. Green: live cells stained with carboxyfluorescein succinimidyl ester (CFSE); red: dead cells stained with propidium iodide (PI); and white: β1,3-,1,4-glucans stained with calcofluor white. Scale bar, 20 µm. (**f**) Comparative analysis of biofilm components. Each quantitative value indicates analyte per gram of towel. The dashed lines on the colony-forming unit (CFU) plots indicate the lower limit of detection (2.78 Log(CFU/g)). *p < 0.05, **p < 0.01, and ***p < 0.001.

To clarify how the towel conditions changed with six months of use, the used towels were collected and compared. Dullness was observed even after 2 months. More dull towels were found after 4 or 6 months (Fig. 2a). Dullness was also observed in the recontaminated towels at 2, 4, and 6 months (Supplementary Fig. 2a). Next, we quantitatively compared the dullness. Using a 12 cm square piece of towel, L*, a*, and b* values were measured with a portable colourimeter, and whiteness (W) values were calculated. In addition, we defined Δ whiteness (Dw) values that indicate how much the towel was discoloured with use and washing.

Changes in whiteness with use, washing, and drying were defined using the following formula:

$$Dw1:\, (W\, value \,of \,new\, towel) - (W\, value\, of \,used \,towel)$$

Changes in whiteness with use only were defined using the following formula:

$$Dw2: \,(W\, value\, of\, recontaminated\, towel) - (W\, value\, of\, used\, towel)$$

Changes in whiteness with washing and drying were defined using the following formula:

$$Dw3:\, (W\, value\, of\, new \,towel) - (W\, value \,of \,recontaminated\, towel)$$

At first, to clarify if it was possible to perform accurate colour measurements with the bumpy structure of the towels, we compared and confirmed that the duller the towels became, the more the value of Dw1 increased. For some towels with a flattened pile structure that have been pressed down with repeated use, Dw1 had negative values (Fig. 2b). Then, the changes in whiteness in each collection month were compared. Dw1 and Dw3 significantly increased in the fourth- and sixth-month-collected towels compared with the second-month collected towels (Fig. 2c, pairwise *t*-test, Dw1: 2–4; *p* = 4.0 × 10^–3^, 2–6; *p* = 4.7 × 1 0^–5^, Dw3: 2–4; *p* = 3.0 × 10^–3^, 2–6; *p* = 8.6 × 10^–5^). Dw2 significantly increased only between second- and sixth-month-collected towels (Fig. 2c, pairwise Wilcoxon test, Dw2: 2–6; *p* = 4.2 × 10^–2^).

Next, the collected towels were microscopically observed to assess the association of microbial adhesion to towels with the confirmed dullness. Microorganisms were stained with carboxyfluorescein succinimidyl ester (CFSE) and propidium iodide (PI) and fibres were stained with calcofluor white (CFW). The results for household D, where the most typical images were obtained, are shown in Fig. 2d,e. Aggregation of microorganisms was not found in the pile structure but only in the ground warp structure. Because the aggregated microorganisms indicated biofilm formation, four typical biofilm components of extracellular polymeric substances (EPS), including polysaccharides, nucleic acids, proteins, and viable cells, were extracted from towel pieces and quantified (Fig. 2f). For EPS, all the measured components increased over time (pairwise Wilcoxon test, polysaccharide: 4–6; *p* = 2.8 × 10^–2^, 2–6; *p* = 3.1 × 10^–2^. Nucleotide: 4–6; *p* = 1.0 × 10^–2^, 2–6; *p* = 3.2 × 10^–4^. Protein: 2–4; *p* = 4.4 × 10^–4^, 4–6; *p* = 2.0 × 10^–3^, 2–6; *p* = 4.9 × 10^–6^). Next, the viable cells in the biofilms were examined by counting the colonies formed when the extracted microorganisms were cultured in potato dextrose agar (PDA) and soybean casein digest agar (SCDA) (Fig. 2f). In both cases, the viable counts significantly increased in towels collected at 6 months compared with those collected at 2 or 4 months (pairwise Wilcoxon test, PDA: 4–6; *p* = 2.5 × 10^–4^, 2–6: *p* = 1.3 × 10^–4^, SCDA: 4–6; *p* = 5.0 × 10^–5^, 2–6; *p* = 6.0 × 10^–3^). In the recontaminated towels, only the proteins and viable counts in PDA increased from the second to the sixth month (Supplementary Fig. 2b).

### Fluctuations in odour intensity and character

**Figure 3:**
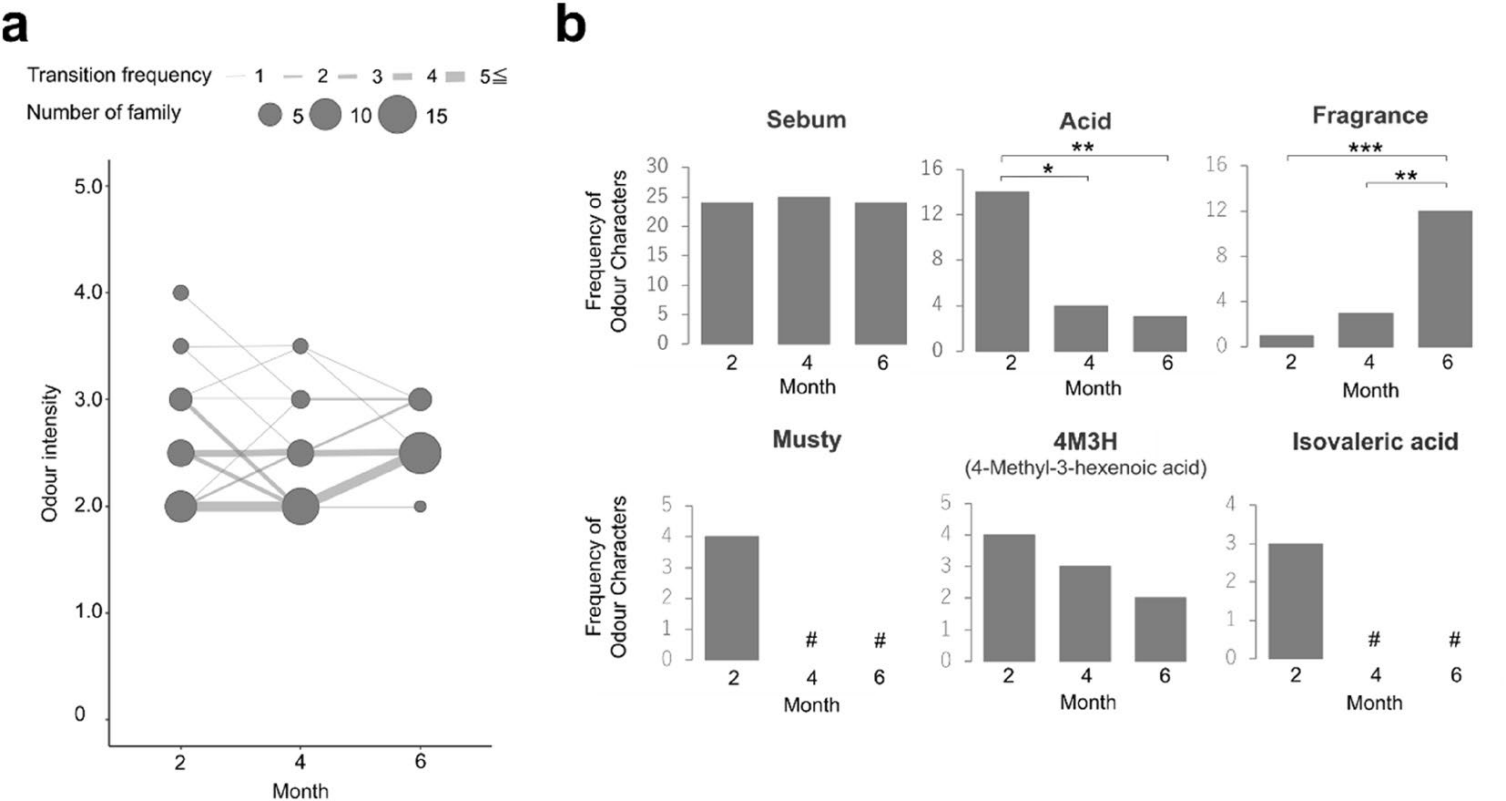
Changes in odour intensity and frequency of each odour type in the used towels. (**a**) Changes in odour intensity in the used towels. Circle size and line width indicate the number of the used towels, and lines indicate the progression of odour intensity of the towels at each time point, from 2 to 4 months and from 4 to 6 months within each household. (**b**) Frequencies of detected odour types. *p < 0.05, **p < 0.01, ***p < 0.001, and # not detected.

In addition to dullness in the collected towels, we conducted sensory tests by trained evaluators to investigate the overall odour intensity and type (Fig. 3). First, the odour intensity of the collected towels was scored on a scale of 0 to 5 in increments of 0.5, which ranged from 2.0 to 4.0. The average odour intensity of the used towels collected at 6 months was higher than that of the used towels collected at 4 months (*p* = 2.4 × 10^–2^). However, the maximum odour intensity decreased over time from 4.0 at 2 months to 3.5 at 4 months and to 3.0 at 6 months (Fig. 3a). Next, we evaluated the odour types of the towels for each collection month (Fig. 3b). In the used towels, sebum odour was detected in more than 20 households in all the collected months. Meanwhile, an acid odour was detected in 14 households at 2 months, which was significantly higher than that in the other collected months (pairwise McNemar test, 2–4; *p* = 1.2 × 10^–2^, 2–6; *p* = 6.8 × 10^–3^). In addition, a fragrant odour was detected in 12 households at 6 months, which was significantly higher than that in the other collected months (pairwise McNemar test, 4–6; *p* = 1.2 × 10^–2^, 2–6; *p* = 1.6 × 10^–3^). Musty and isovaleric acid odours were detected only at 2 months in four and three households, respectively. The odour of 4-methyl-3-hexenoic acid (4M3H), which causes a ‘wet and dirty dust-cloth-like’ odour^15^, was detected in less than five households in all the collected months. The used and recontaminated towels were also compared. The used towels had higher odour intensity than the recontaminated towels at all time points. Additionally, no odour type was specific to the recontaminated towels (Supplementary Fig. 3).

### Microbiome analysis of towel biofilms

**Figure 4:**
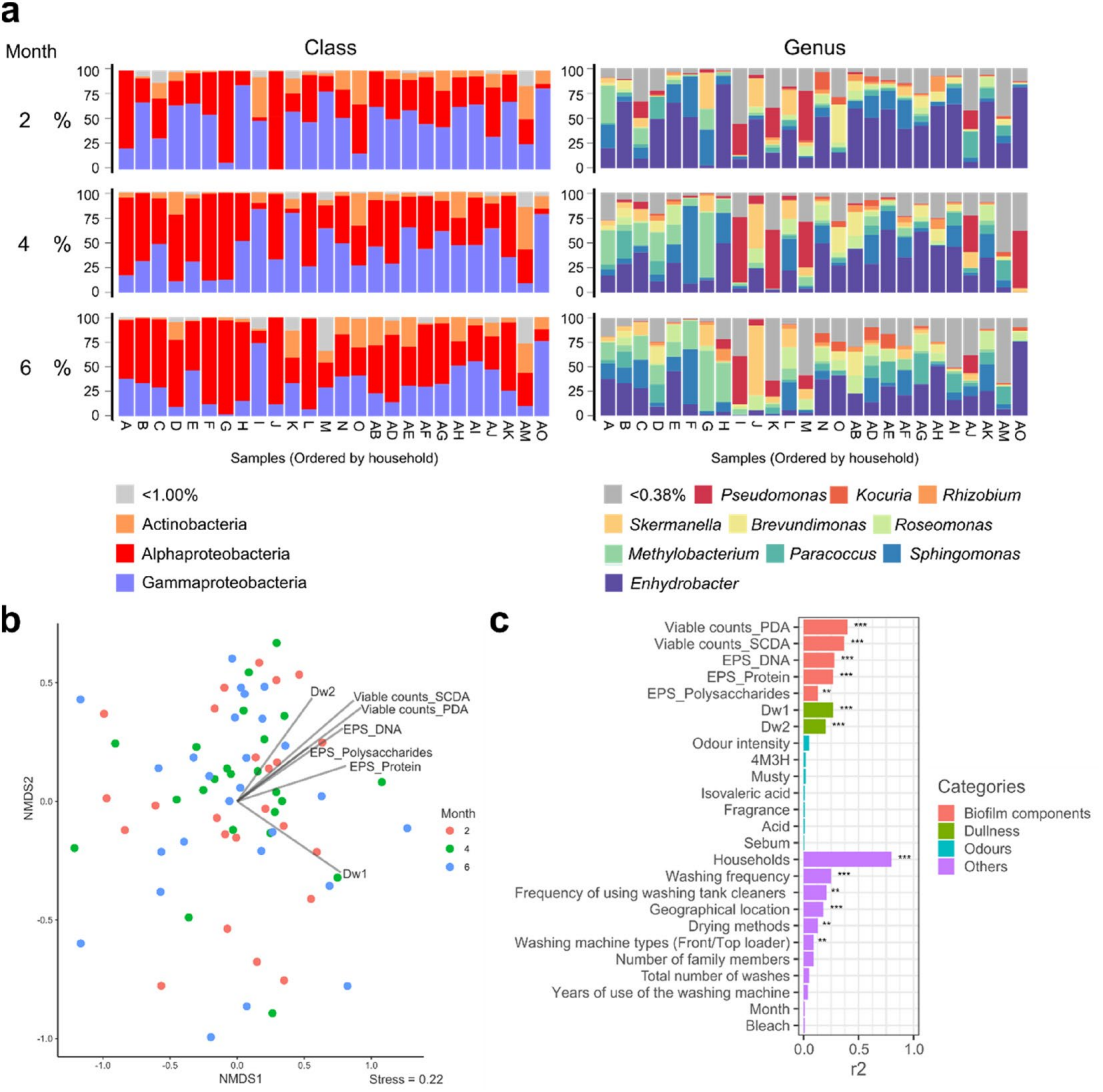
Microbiota in the used towels and the association with various metadata. (**a**) Microbiota in the used towels. Each bar displays the relative abundance of species at class (left) and genus (right) levels. Species with high median relative abundances (left: > 1%, right: > 0.38%) are shown as coloured bars. The remaining species (left: median relative abundance < 1%, right: median relative abundance < 0.38%) are shown as a single grey bar. (**b**) NMDS and envfit results. Seventy-one sample plots are ordinated in NMDS space based on Bray–Curtis dissimilarities calculated from operational taxonomic unit abundance tables. Vectors with significant correlations (*p* < 0.001) are fitted using envfit and the length of the arrow is proportional to the strengths of the correlations. (**c**) Variance (r^2^) explained by each covariate in the model determined by envfit. Covariates are coloured based on the metadata category. *p < 0.05, **p < 0.01, and ***p < 0.001.

We investigated the bacterial species localised on the towels and how they are related to factors such as cleaning habits. The biofilms between the fibres of the ground warp of the towel appeared to be hardly extracted by physical and chemical treatment during DNA extraction because the bacteria were protected by the towel structure. Thus, we investigated various DNA extraction methods. When DNA extraction was attempted using sodium dodecyl sulphate or other detergents, bacteria adhering to the towel remained even after the extraction. Even after freeze-crushing, the bacteria remained on the fibres. When physical crushing using beads was combined with solubilisation using phenol:chloroform:isoamyl alcohol (25:24:1), no bacteria remained after DNA extraction (Supplementary Fig. 4). Next, as the biofilms on towels were thought to be localised, the optimum towel size for bacterial analysis was investigated. Extraction of biofilms from 1 cm square pieces of towels showed similar microbial compositions to those in the surrounding area (Supplementary Fig. 5). Therefore, this sample size was employed for microbiome analysis. To verify the validity of the processes from DNA extraction and purification to the preparation of the sequencing library, a 1 cm square piece of the new towel with added cell mock community (Biological Resource Center, National Institute of Technology and Evaluation; Cell-Mock-001) was analysed using the aforementioned method. In addition, to verify the validity of the preparation of the sequencing library only, a DNA mock community (Biological Resource Center, National Institute of Technology and Evaluation; DNA-Mock-001) was analysed using the same method. We confirmed that all the included bacterial species in each mixture were detected as expected abundances (Supplementary Fig. 6). We then used our optimised DNA extraction method to analyse the collected towels. For the used towels, the detected bacteria were sorted at the class level, and the major bacteria belonged to Gammaproteobacteria, Alphaproteobacteria, and Actinobacteria (median relative abundances, > 1.00%). At the genus level, *Enhydrobacter*, *Sphingomonas*, *Paracoccus*, *Methylobacterium*, *Roseomonas*, *Brevundimonas*, *Skermanella*, *Rhizobium, Kocuria*, and *Pseudomonas* were detected at relatively high abundances (median relative abundances, > 0.38%). In contrast, major bacteria comprising the Japanese skin microbiota, *Cutibacterium*, *Staphylococcus* and *Corynebacterium*, were detected at relatively low abundances (median of 0.08% for *Cutibacterium*, 0.04% for *Staphylococcus* and 0.04% for *Corynebacterium*; Fig. 4a).

Next, we examined how the microbiota differed by sampling month using the Bray–Curtis dissimilarity index at the genus level. The obtained values were plotted in two-dimensional space by non-metric multidimensional scaling (NMDS). The data were dispersed in two-dimensional space, with no cluster according to sampling month (Fig. 4b). Next, indirect gradient analysis was performed to investigate which factors are related to the microbiota. Metadata for analysed subjects are provided in Supplementary Table 1. We observed the correlations with all biofilm components, including EPS, viable counts, and dullness intensity. In addition, the correlations with household, washing frequency, frequency of using washing tank cleaner, geographical location, drying methods, and washing machine types (front-loading or top-loading) were detected (Fig. 4c). Of these, the continuous metadata variables with *p* < 0.001 were described as vectors (Fig. 4b). Furthermore, when the same Bray–Curtis dissimilarity indices were plotted in three-dimensional NMDS (stress value = 0.16), a significant correlation (*p* < 0.05) was also found for the same metadata as that in two-dimensional NMDS. Next, we confirmed if the microbiota changes with or without towel use. We plotted the microbiota data obtained from the used and recontaminated towels in a two-dimensional space and performed an indirect gradient analysis, which showed that towel use was not correlated with the microbiota (*p* = 9.8 × 10^–1^, r^2^ = 1.0 × 10^–4^).

### Network analysis

**Figure 5:**
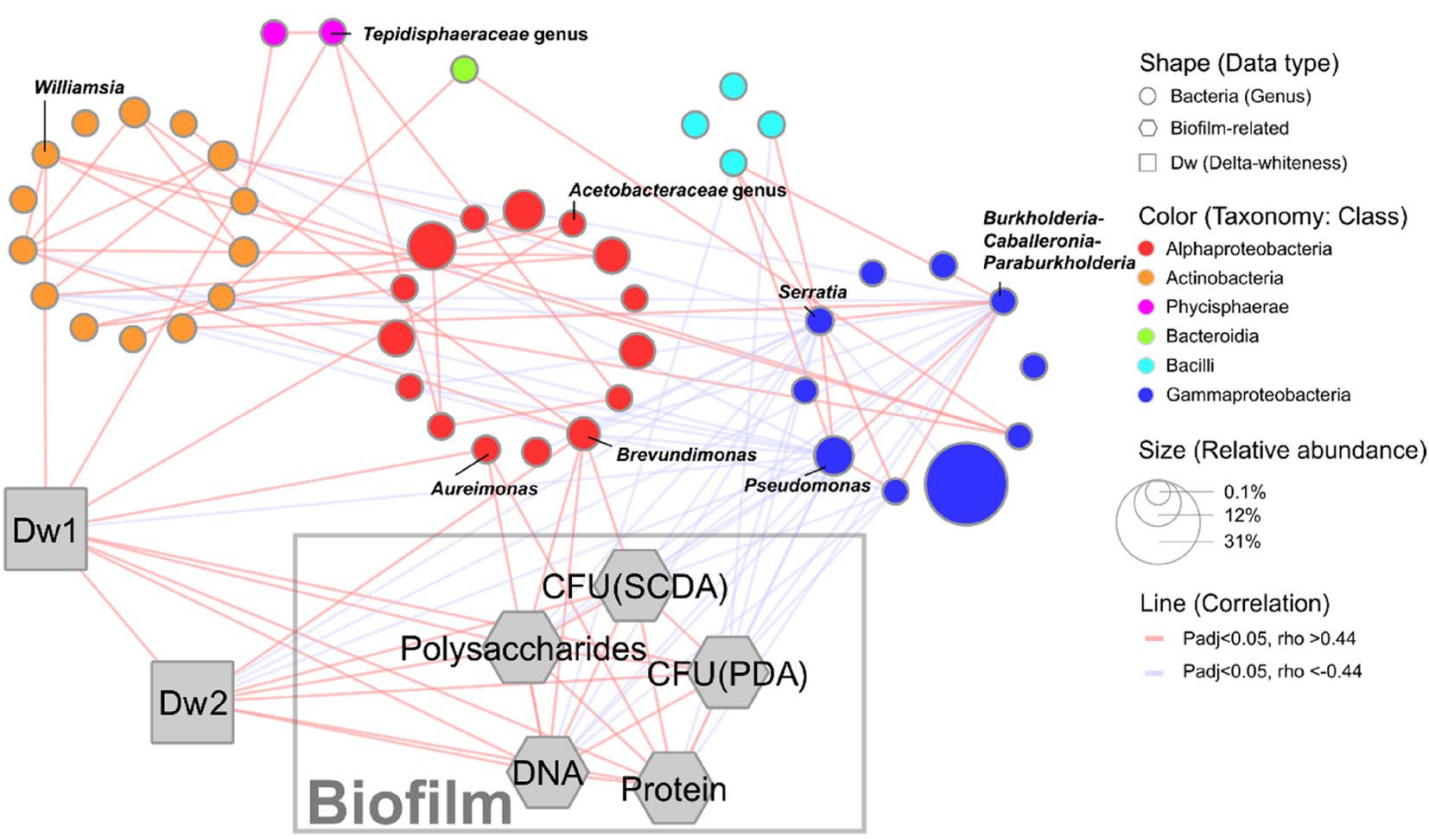
Network analysis of bacterial species, dullness, and biofilm components. The nodes indicate genera with mean relative abundance ≥ 0.1% and associated metadata. The node shape indicates the data type. Circles indicate genera, which are coloured by their class, and the size indicates the relative abundance. Lines indicate correlations (*p* < 0.05). Red and blue lines indicate positive and negative correlations, respectively. Grey hexagons indicate the biofilm components and grey squares indicate the Δ whiteness (Dw) value.

To deeply analyse the relationship between the microbial abundances of towel samples and those with other acquired data, Spearman’s rank correlation tests were performed for bacterial genera detected at mean relative abundances of > 0.1%, dullness, and biofilm components. The factors that were significantly correlated (*p* < 0.05) and had highly significant correlation coefficients (|ρ|> 0.4) were connected by a line (Fig. 5). Regarding dullness, positive correlations were observed between Dw1 and the biofilm components except for viable counts when the microorganisms were cultured in the SCDA medium. Positive correlations were also found between Dw2 and all biofilm components. Further, the biofilm components except for viable counts when the microorganisms were cultured in the PDA medium were positively correlated with *Aureimonas* or *Brevundimonas*, which belong to Alphaproteobacteria. In addition, all the biofilm components showed negative correlations with some bacteria belonging to Bacilli or Gammaproteobacteria. Bacteria that showed a direct positive correlation with dullness intensity were *Aureimonas*, *Acetobacteraceae* genus, *Tepidisphaeraceae* genus, and *Williamsia* in Dw1, while only *Brevundimonas* was positively correlated with dullness in Dw2. Several bacteria showed a negative correlation with dullness intensity. For Dw1, only *Pseudomonas* was negatively correlated with dullness, while *Burkholderia*, *Pseudomonas*, and *Serratia* negatively correlated with dullness in Dw2. The relationship between microbial abundances from the recontaminated towels and the dullness intensity of the towels was analysed by Spearman’s rank correlation tests in the same manner. As a result, the bacterial species positively correlated with Dw3 were *Sphingomonadaceae* (ρ = 0.47, *p* = 1.8 × 10^–2^) and *Aureimonas* (ρ = 0.46, *p* = 2.8 × 10^–2^).

## Discussion

There are many reports regarding biofilm formation in textile environments, particularly on odour generation in garments such as T-shirts^10–12^. The microbiota of textile products used in daily life has previously examined^6^; however, biofilm formation on textile products used in daily life has not been reported so far. This is the first study to investigate biofilm formation in towels in daily life, which are important for hygiene and unique in terms of fibre structure and usage. We analysed these biofilms from various perspectives, including towel laundering habits. Our results provide a detailed understanding of biofilms unique to towels, helping us to consider relationships between microbial composition and dullness or odour and to develop microbial control guidelines in towels.

Among the various perspectives, dullness was regarded as a characteristic feature because many towels were clearly discoloured, and the dullness increased significantly at 6 months. (Fig. 2c, Dw1). This dullness may have been generated on the fibre surface through towel use or transferred from other clothes or washing machines while laundering. It is quite natural to consider that dullness was caused by use, but we also investigated the transfer of dullness while washing. To explore this transfer, we first evaluated recontaminated towels that were not used but washed with used towels. As a result, dullness from both use (Dw2) and other than use (Dw3) increased over time. This suggests that towels can be discoloured by both towel use and the transfer of soils during washing. Grime, mud, and other particles that adhered during towel use may remain on the towel even after washing (Fig. 2c). In microscopic analysis to investigate the dullness in detail, biofilm-like structures were observed 2 months after the start of use and appeared to increase as the degree of dullness increased (Fig. 2e). Previous reports^9,16^ mention biofilms observed on textile products. However, all the reported biofilms were formed by inoculating bacteria on textile products and by culturing the bacteria in the laboratory; hence, the time to form biofilms and their characteristics were investigated. In this report, we observed biofilms in textile products used in daily life for the first time and showed that biofilms could keep forming over the six-month period in towels. In fact, the EPS amount and viable counts increased over time (Fig. 2f), which was consistent with the microscopic observation (Fig. 2e). These data suggest that towels could get discoloured as biofilms developed during six months of use. It should be noted that biofilms in textile products have been generally evaluated by the number of live bacteria extracted from the yarn^16^, but we also used EPS quantification to evaluate the biofilm amounts. Compared with other methods such as measuring biovolume using microscopy^1^, EPS quantification is more suitable for capturing the characteristics of the entire biofilm present on textiles, as it allows for a more comprehensive quantification of biofilms. The quantitation enabled a precise evaluation of biofilms as well as various comparative analyses among the microbial abundances and biofilm components. For example, in the recontaminated towels, unlike the used ones, only the viable counts on PDA medium and the protein amount increased over time among the biofilm components. This is possibly because the composition of the biofilms in the used towels and that in the recontaminated towels might be different. Although additional long-term investigations are needed, some biofilm components may contribute to the balance of microbial adherence and their transition to the fibre structures. However, there are other possibilities, such as relatively low biofilm amounts in the recontaminated towels that make it difficult to detect the changes in constituent amounts over time.

In addition to dullness, we also focused on odour types and intensity, as several studies have reported their association with the microorganisms in textile products^10–12^. Regarding odour types, the acid odour was significantly more frequent at 2 months than at other collected points, and the fragrant odour was significantly more frequent at 6 months (Fig. 3b). Acid odour is a type of ‘wet and dirty dust-cloth-like’ odour whose main component is short-chain fatty acids. The collection time at 2 months for this study was in July, which is when temperature and humidity are high in Japan and ‘wet and dirty dust-cloth-like’ odour is likely to occur. Acid odours were likely seasonal and may be affected by changes in the microbiota and the metabolic conditions of bacteria. Furthermore, the beta-oxidation of sebum and the state of textile coatings may also be involved in the development of acid odour. In contrast, fragrant odours arising from fragrances contained in fabric softeners and detergents were high at 6 months. Thus, it was possible that the fragrant components accumulated on the towel over time, making it easier to detect. In addition, sebum odour was detected in almost all towels at all the collected points. This suggests that sebum removal during laundry might not be enough and might not be related to biofilm formation. From these results, it is possible that the seasonal ‘wet and dirty dust-cloth-like’ odour detected at 2 months disappeared over time, residual sebum would have been oxidised, and fragrant components would have accumulated over time, resulting in higher frequent sebum and fragrant odour. Therefore, significantly higher odour intensity might be detected at 6 months compared to 4 months (Fig. 3a). However, to verify the above possibilities, it is necessary to evaluate not only the entire odour intensity but also the odour intensity for each odour type. In addition, detected odour types from the recontaminated towels were the same as used towels (Supplementary Fig. 3c). Therefore, the occurrence of odour may be affected by seasonal fluctuations, bacterial species, or soil types rather than towel usage patterns. Odour intensity in the used towels was significantly higher than that in the recontaminated towels at all collected points. Thus, a larger amount of biofilm components may lead to increased odour component adhesion and accumulation.

To consider the relationships among dullness, odour, and microorganisms, it is necessary to accurately recover bacterial DNA from the biofilms. The bacterial cells attached to fibres were observed, even after being extracted according to a previous method^5^. Finally, by suspending the fibres in phenol:chloroform:isoamyl alcohol (25:24:1) in addition to freeze-crushing, bacterial cells were no longer observed (Supplementary Fig. 4). Thus, biofilms might adhere strongly to towels. In the results of microbiome analysis, the proportions of *Cutibacterium*, *Staphylococcus* and *Corynebacterium*, which are the main components of skin microbiota, were relatively low (Fig. 4a). Bacteria that make up the skin microbiota adhere to clothing such as T-shirts^6^, and they are also the main components of microbiota in garments, since clothing is in constant contact with the skin. However, towels are in contact with the skin only when in use. In addition, the unique fibre structure of towels, different from other clothing, might inhibit bacterial desorption by washing. Furthermore, the bacterial species that grow on towels could differ depending on the temperature and humidity, which are different from the conditions on human skin.

When the correlations between microbial abundances, biofilm components, dullness, odour, or laundry habits were analysed, biofilm components and dullness correlated with microbial composition (Fig. 4c), which suggests that a unique microbial composition may be involved in biofilm formation and towel dullness. In terms of the correlation with laundry habits, the microbial abundances correlated with the households but not with the collected month. Thus, each household was thought to have unique towel microbiota. What caused the uniqueness of the microbiota is unclear and further analysis is required, but certain factors were shown to correlate with the microbiota, including washing frequency, frequency of using washing tank cleaner, experimental location, drying methods, and washing machine types (Fig. 4c). This suggests that laundry habits are important factors influencing the towel microbiota; in addition to the items investigated in this study, other factors such as inlet water quality of the households, washing machine microbiome, drying conditions and detergent use may affect biofilm formation and dullness. On the other hand, no significant differences were observed in the microbial composition of the used and the recontaminated towels, suggesting that use may not significantly affect microbiota in towels.

We further analysed the correlations with more specific constituent bacterial genera (Fig. 5). *Aureimonas* and *Brevundimonas* showed a positive correlation with the amount of biofilm components and dullness intensity. These bacterial genera belong to Alphaproteobacteria and are detected in environments such as the rhizosphere, phyllosphere, and skin. Although these genera can be detected on the skin, these genera have never been reported as constitutive bacteria of skin to our knowledge. Therefore, our results suggest that some bacterial species would be more suited to the fibrous towel environment and predominantly form biofilms. Regarding the adhesion step of the biofilm formation, many Alphaproteobacteria were reported to concentrate polysaccharides on one side of the cells and use these polysaccharides to attach to abiotic surfaces^17^. For example, *Brevundimonas subvibrioides* forms a complex of polysaccharides called holdfast at one end of the cell and attaches this structure to abiotic surfaces^18^. The closely related marine bacterium *Hirschia baltica* was reported to adhere to abiotic surfaces even under high ionic strength by adjusting the holdfast structure via polysaccharide deacetylation^19^. Some *Brevundimonas* strains have a gene homolog of the deacetylase^20^. Thus, *Brevundimonas* could be efficiently attached to the yarn even in a concentrated high-ion environment with concentrated residual soils, detergents, and other components. Although the mechanism by which *Aureimonas* forms biofilms has not been reported in detail, many closely related bacteria that belong to *Rhizobiales* attach to plants using unipolar polysaccharides^21^. Thus, *Aureimonas* might attach to cellulose fibres in towels using a similar mechanism. Both bacteria could adhere to the towel more strongly than other bacteria and form biofilms using these specialised adhesion mechanisms discussed above. In the future, the phenotypic analysis of the bacteria on the fibres would help understand the mechanism of biofilm formation on towels and dullness related to biofilm formation. In any case, the microbial composition was significantly different from that of general skin microbiota, which indicates that bacterial transfer from the skin would not have a large effect on the microbial composition of towels. Rather, the bacteria that are more strongly compatible with the towel yarn could predominate, although the bacteria within the towels are likely to originate from diverse environments such as human skin, washing water, airborne dust and dirt, and soils in the laundry environment. Our study shows the complexity of textile product environments as microbial habitats. To understand bacteria in textile environments, it will be necessary to consider not only the fibre materials, but also laundry habits, structures of textiles, usage patterns, physiological features and topographical features.

## Methods

### Study overview

The study adhered to the guidelines of the Declaration of Helsinki. Also, no personal data of the towel users were recorded, rendering it impossible to assign a specific data to a specific user afterwards. Moreover, the towel users did not provide any directly health-related personal data, and the analyses were not aimed at the detection of directly health-related bacteria, such as obligate pathogens. The Japanese Ministry of Health, Labour and Welfare (MHLW) guidelines “Ethical Guidelines for Life Science and Medical Research Involving Human Subjects” stipulate that “research does not constitute ‘human subjects’ research if it does not involve events related to human health”, and the Human Research Ethics Committee, Kao Corporation, which follows this guideline, considered that ethical review is not required for this research.

After obtaining informed consents from the participants, the survey was conducted at 26 households in the Wakayama and Tochigi prefectures in Japan. Six new cotton towels (33 cm × 73 cm) were prepared for each household. Red tags were sewn on three towels and blue tags were sewn on the remaining three towels. The towels of each colour were numbered. Red-tagged towels were freely used and washed. Blue-tagged towels were only washed with red-tagged towels with the same number (recontaminated towels). Corresponding red- and blue-tagged towels were collected every 2 months after the start of use. A 12 cm square piece was cut out from a frequently used site using a cutter cleaned with 70% ethanol. The piece was separated as needed and we performed EPS, viable cell counts, and microbiota analysis based on 16S rRNA, in addition to the analysis of odour intensity, odour species, and dullness intensity. Finally, we collected information on laundry habits in a questionnaire.

### Measurement of dullness intensity

The lightness value (L*), red/green value (a*), and blue/yellow value (b*) were measured with a portable colour difference meter (NF777, Nippon Denshoku) using a 12 cm square piece of towel. The whiteness was calculated from the obtained values^22^. Areas from the same towel piece were measured four times, and the average value was considered the whiteness (W) value. Next, we calculated Δ whiteness (Dw) values to show how much the towel was discoloured by use and washing. The definition and calculation of each value are shown below.

Changes in whiteness with use, washing, and drying were defined using the following formula:

$$Dw1: \,(W\, value\, of \,new\, towel) - (W \,value \,of\, used\, towel)$$

Changes in whiteness with use only were defined using the following formula:

$$Dw2:\, (W \,value \,of\, recontaminated \,towel) - (W\, value\, of\, used\, towel)$$

Changes in whiteness with washing and drying were defined using the following formula:

$$Dw3:\, (W\, value\, of\, new\, towel) - (W\, value\, of\, recontaminated\, towel)$$

### Odour evaluation

A 5-cm square piece of each towel was used to evaluate the odour type and intensity as described in a previous report^15^. The odours were assessed by five or more specialised evaluators who received training in odour assessment for each odour character. When five or more of these evaluators detected the presence of any type of odour, the towel was judged to have an odour. The overall odour intensity was evaluated using six grade indicators: 0 (odourless), 1 (very faint), 2 (faint), 3 (medium), 4 (strong), and 5 (very strong).

### Microscopy

Towels were imaged using a confocal laser scanning microscope (LSM880, Zeiss). For live bacteria, 10 mg/mL Cellstain-CFSE (Dojindo, Ex: 488 nm, Em: 490–606 nm) was dissolved in dimethyl sulfoxide (Fujifilm Wako). Staining was performed at a final concentration of 20 µg/mL. Dead bacteria were stained with 1.0 mg/mL Bacstain-PI solution (Dojindo, Ex: 561 nm, Em: 568–707 nm) at a final concentration of 2.0 µg/mL. Cotton fibres were stained with 1.0 mg/mL Calcofluor White M2R (CFW, Merck, Ex: 405 nm, Em: 410–497 nm) dissolved in saline at a final concentration of 20 µg/mL.

### DNA extraction

From the original towel samples, a 1-cm square was divided into four to make 0.5-cm squares, which were individually placed into 2-mL volume freezing and crushing tubes (Yasui Kikai) with a metal cone (Yasui Kikai). The tubes were placed in liquid nitrogen, and the samples were crushed twice with a multi-bead shocker (Yasui Kikai) at 2,000 rpm for 1 min. A 1 mL volume of buffer (10 mM EDTA, 1.0% (w/v) sodium dodecyl sulphate, and 10 mM Tris–HCl, pH 8.0) was added to the crushed tube and stirred vigorously. A 500 µL volume of the supernatant containing crushed fibres was collected from each of the four tubes and transferred to a clean tube. A 700 µL volume of phenol:chloroform:isoamyl alcohol (25:24:1) and zirconia beads (Zircoprep Mini, Nippon Genetics) were added to each tube. The samples were then vigorously stirred for 5 min. After centrifuging at 8000 × *g* at 25 °C for 10 min, the aqueous layer was transferred to a new tube. A 700 µL volume of phenol:chloroform:isoamyl alcohol (25:24:1) was added to the tube and stirred vigorously for 5 min. The tube was centrifuged at 13,800 × *g* for 5 min at room temperature. A 400 µL volume of the aqueous layer was then transferred to a new 1.5-mL centrifuge tube. Genomic DNA was purified using Ethachinmate (Nippon Gene). The samples were air-dried and lysed in 100 µL TE buffer (pH 8.0) (Nippon Gene). The prepared genomic DNA was diluted tenfold with TE buffer (pH 8.0) to reduce the effect of PCR inhibitors (e.g. polysaccharides and metal ions) and then used for amplicon sequencing of 16S rRNA genes, as described in the following section.

### Measurement of biofilm components

We quantified extracellular polysaccharides, proteins, and DNA. A 30-mg sample of the cut towel pieces was suspended in 1 mL 0.1 M NaOH, heated at 100 °C for 3 h, and centrifuged at 13,800 × *g* for 5 min and the supernatant was collected and used for the subsequent analysis. The extracellular polysaccharides were quantified using the phenol sulphate method^23^. The proteins were quantified using the Lowry method^24^, and the double-stranded DNA was quantified using a Qubit^®^ 3.0 Fluorometer (Thermo Scientific).

The viable cell counts were determined from a 0.5 cm square towel piece. Each sample was mixed with 2.0 mL Lecithin Polysorbate (LP) diluted solution (Fujifilm Wako) and sonicated for 10 min. Then, it was streaked and cultured on SCDA medium (SHIOTANI M.S.) for bacteria and PDA medium (Becton Dickinson) for bacteria and fungi. The number of colonies was counted after culturing at 37 °C for 6 days for SCDA medium and 30 °C for 6 days for PDA medium.

### Amplicon sequencing of 16S rRNA genes and data analysis

Sequencing libraries were prepared using a modified Illumina manufacturer’s protocol (https://support.illumina.com/content/dam/illumina-support/documents/documentation/chemistry_documentation/16s/16s-metagenomic-library-prep-guide-15044223-b.pdf) as follows. The V3V4 region of 16S rRNA genes was amplified using primers 341F: 5ʹ-CCTACGGGNGGCWGCAG-3ʹ^25^ and 805R_mod: 5ʹ-GACTACHVGGGTATCTAAKCC-3ʹ. The reverse primer was designed by replacing the base 805R^25^ sequence with one base to efficiently detect *Cutibacterium* (19 T > K). The libraries were sequenced using a MiSeq Reagent Kit v3 (2 × 301 cycles) on a MiSeq System (Illumina), and PhiX (Illumina) was added to each library pool. Mock communities (Cell-Mock-001 and DNA-Mock-001; NITE Biological Resource Center, National Institute of Technology and Evaluation) were analysed as controls.

The sequencing data were analysed with QIIME 2^26^. The SILVA-138 database^27^ was used for bacterial annotation. All the analyses were performed using R software (version 4.0.2). First, phyloseq package^28^ was used to analyse β-diversity. Accordingly, a rarefaction curve was created (Supplementary Fig. 7), the number of reads considered to be sufficient α-diversity was set to 10,000, and five samples with less than 10,000 reads were excluded. Next, the number of reads in all samples was downsampled to 11,227 reads, which was the lowest read number in all the remaining samples. The obtained data were summarised at the genus level and a two-dimensional distance matrix was created by NMDS using Bray–Curtis dissimilarity as a distance index. The correlation analysis between the microbial composition and each metadata category, such as odour, dullness, and laundry habits (Table S1), in the used towels was performed using the envfit function in VEGAN R package^29^.

The pairwise Spearman correlation analysis was performed using the corr.test function in psych package^30^ for bacteria (genus level) with a mean abundance > 0.1% and each metadata term. The Holm method was used to correct the *p*-values. Cytoscape^31^ was used to visualise the results. The data of the used towels and the recontaminated towels for Spearman’s rank correlation tests are provided in Supplementary Tables 2 and 3, respectively.

In examining the optimal size of the towels for DNA extraction (Supplementary Fig. 5), the same methods were used except for the following points. For the sampling aria, sampling was performed on the four adjacent 1 cm pieces of towels from each household. For rarefaction, the number of reads in all samples was downsampled to 40,000 reads, which was below the lowest read number in all the samples. For beta diversity analysis, a two-dimensional distance matrix was created by principal coordinate analysis, which is considered suitable for a small number of samples, and Bray–Curtis dissimilarities were calculated as the distance, and a heatmap was created using the drawing software ComplexHeatmap^32^.

### Statistical analyses of various metadata

All the statistical analyses were performed using R (version 4.0. 2). The Shapiro–Wilk test was performed to confirm normality of the data of dullness intensity, EPS, viable counts, and odour intensity. The data under a 5% significance level were considered to be non-normally distributed and were analysed using pairwise Wilcoxon tests in rstatix package^33^. The normally-distributed data were analysed with pairwise *t*-tests in rstatix package^33^. For odour species variation, multiple comparison analysis was performed with pairwise McNemar tests in rcompanion package^34^. The Bonferroni method was applied to correct the *p*-values in the multiple comparisons above.

## Supplementary Information

Supplementary Figures.

Supplementary Tables.

## Data Availability

The 16S rRNA gene sequences were deposited in the DDBJ Sequence Read Archive (http://www.ncbi.nlm.nih.gov/bioproject/, Bioproject IDs = PRJDB15097, Run = DRR435712-DRR435889).
