# Supplementary material for: Analysis of biofilm and bacterial communities in the towel environment with daily use
Source: Sci Rep. 2023 May 10;13:7611. doi: 10.1038/s41598-023-34501-4 (PMC10172380; doi:10.1038/s41598-023-34501-4)
Supplement: Supplementary file 1 — Supplementary Figures. [file 41598_2023_34501_MOESM1_ESM.docx]

**Supplementary Information**

**
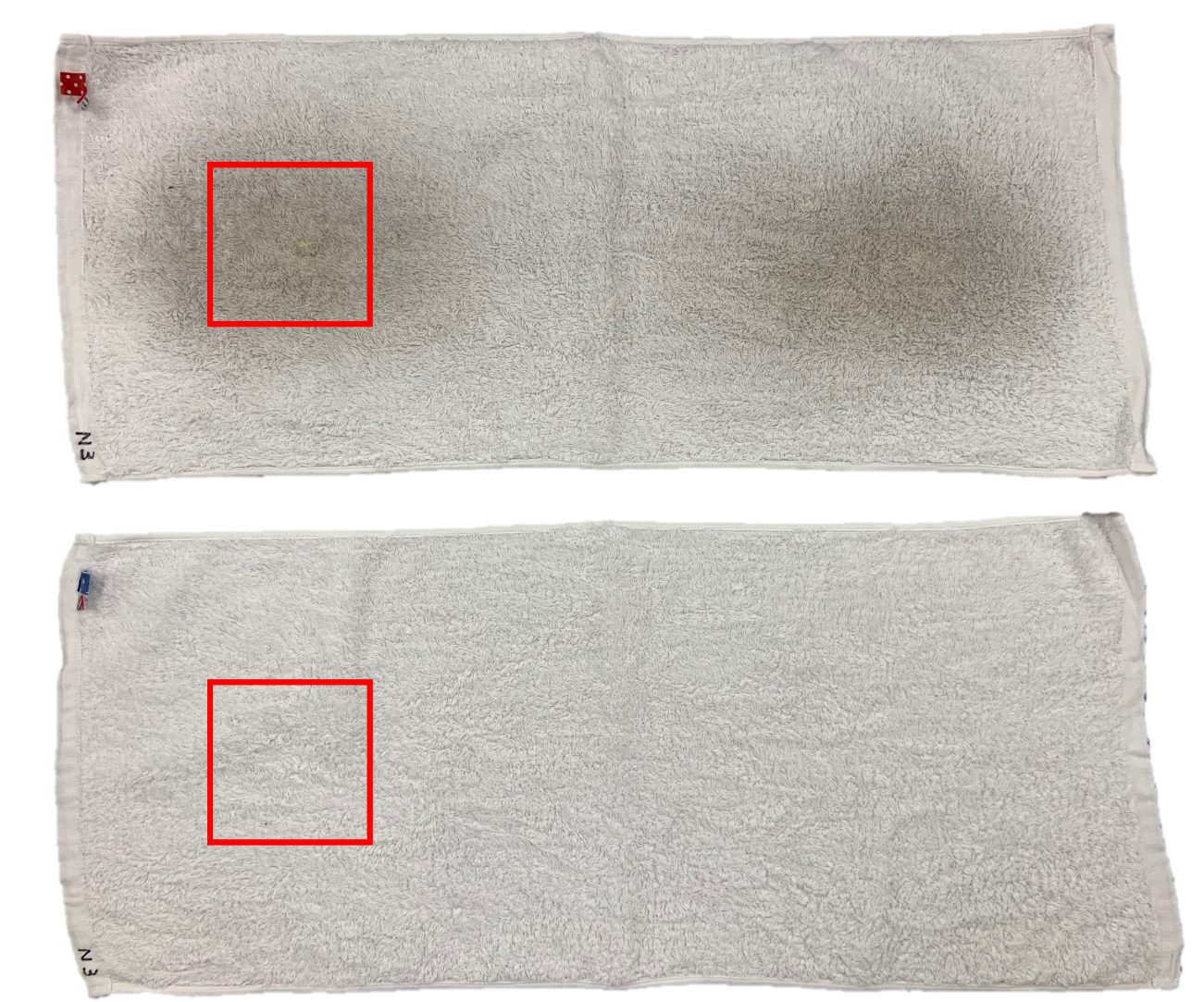
**

**Supplementary Fig. 1** **Collected towels and analysed areas.**

Collected towels from household N at 6 months after distribution; the upper is the used towel and the lower is the recontaminated towel. The red rectangles represent the 12 cm square areas to be cut and analysed.

**
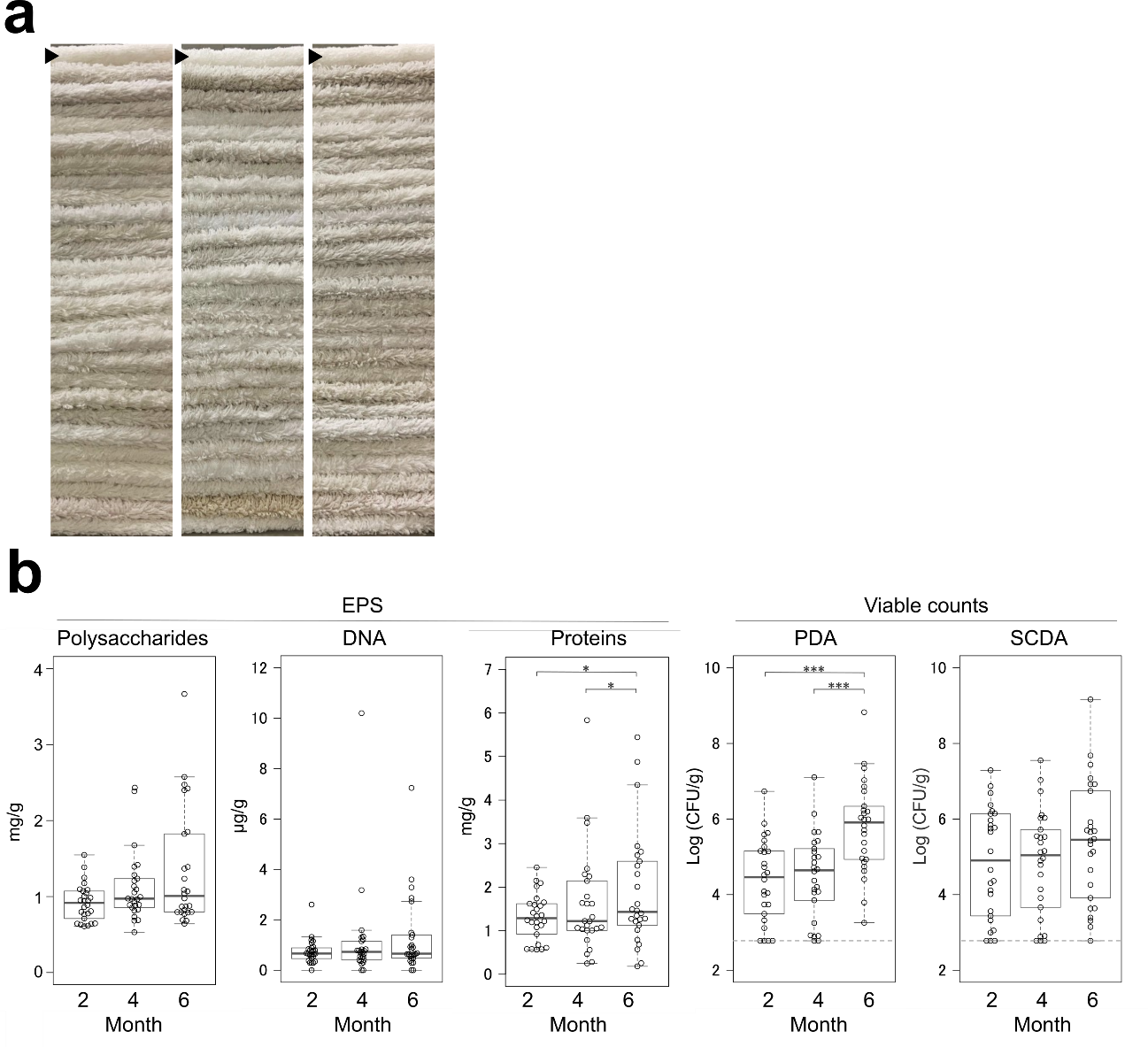
**

**Supplementary Fig. 2 Sequential changes in dullness and biofilm components in the recontaminated towels.**

**a** Images of the collected towels that were not used but washed with the used towels for 2, 4, and 6 months. The towels were stacked and arranged by collected months. Arrowheads indicate towels that were not used or washed. **b** Changes in biofilm components. Each value on the y-axis indicates a quantitative result per gram of towel. The dashed lines on the CFU plots indicate the lower limit of detection (2.78 Log(CFU/g)). **p* < 0.05, ***p* < 0.01, and ****p* < 0.001.

**
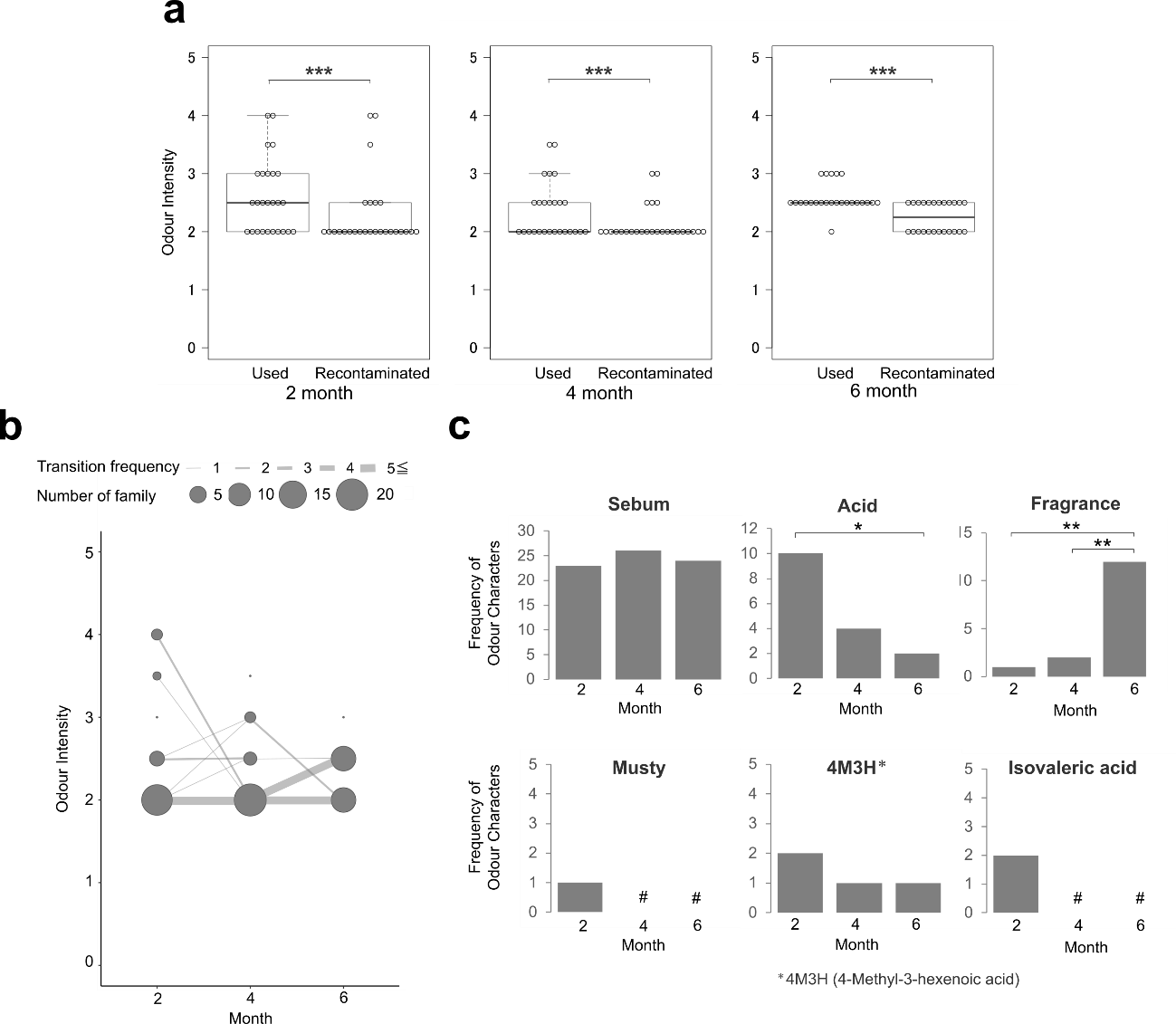
Supplementary Fig. 3 Odour intensity in the used and the recontaminated towels.**

**a** Comparison of odour intensity between the used towels (left) and the recontaminated towels (right). **b** Changes in odour intensity in the recontaminated towels. Circle size and line width indicate the number of the recontaminated towels, and lines indicate the progression of odour intensity of the towels at each time point from 2 to 4 months and from 4 to 6 months within each household. **c** Odour detection frequencies of the recontaminated towels. **p* < 0.05, ***p* < 0.01, ****p* < 0.001, and #not detected.


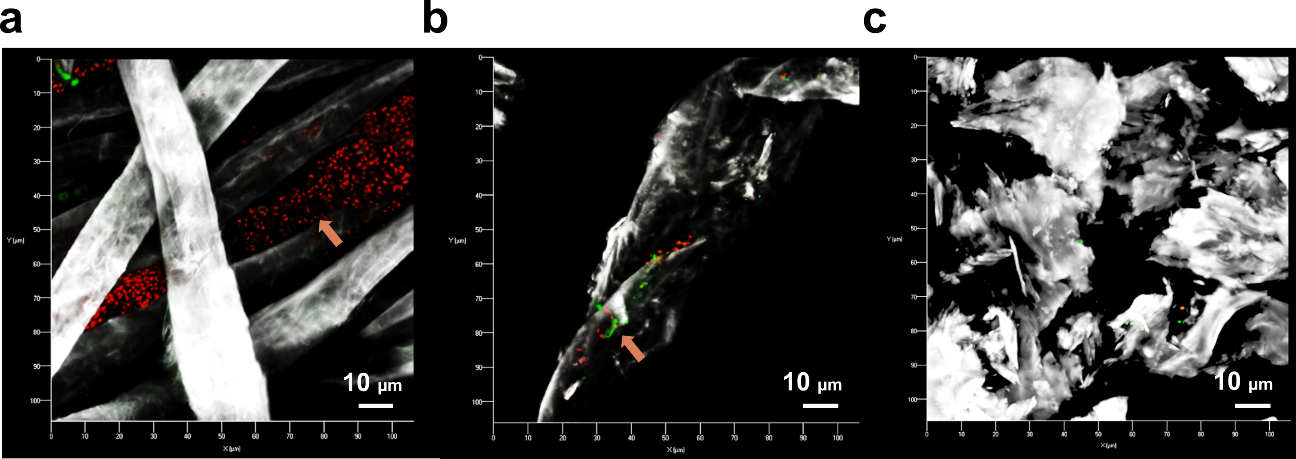
 **Supplementary Fig. 4 Comparison among DNA extraction methods for towels.**

Microscopic images of towels after vortexing and sonication in lysis buffer comprised of EDTA and SDS (**a**), freeze crushing (**b**), and freeze crushing and extraction with phenol:chloroform:isoamyl alcohol (**c**). Green; live cells stained with CFSE, red; dead cells stained with PI, and white; β1,3-,1,4-glucans stained with Calcofluor White.


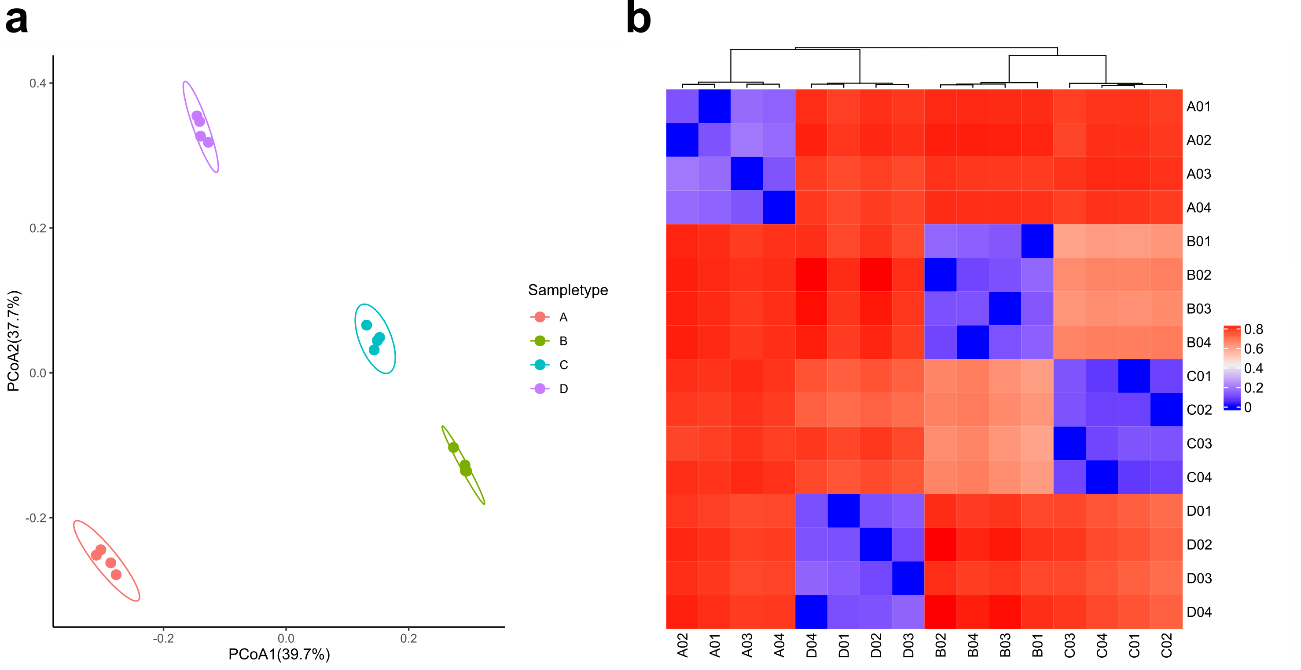


**Supplementary Fig. 5 Bacterial β-diversity from adjacent 1 cm square towel samples.**

Comparison of the β-diversity of four adjacent pieces of 1 cm square samples for four different towels. **a** Principal coordinate analysis based on the Bray–Curtis dissimilarity of microbial communities in each sample. Principal coordinate analysis was used because of the small number of samples. Ellipses show the 95% confidence interval for each towel. **b** Heatmap of the distance matrix was created using software ComplexHeatmap (version 2. 14. 0). Columns were ordered by hierarchical clustering.

**
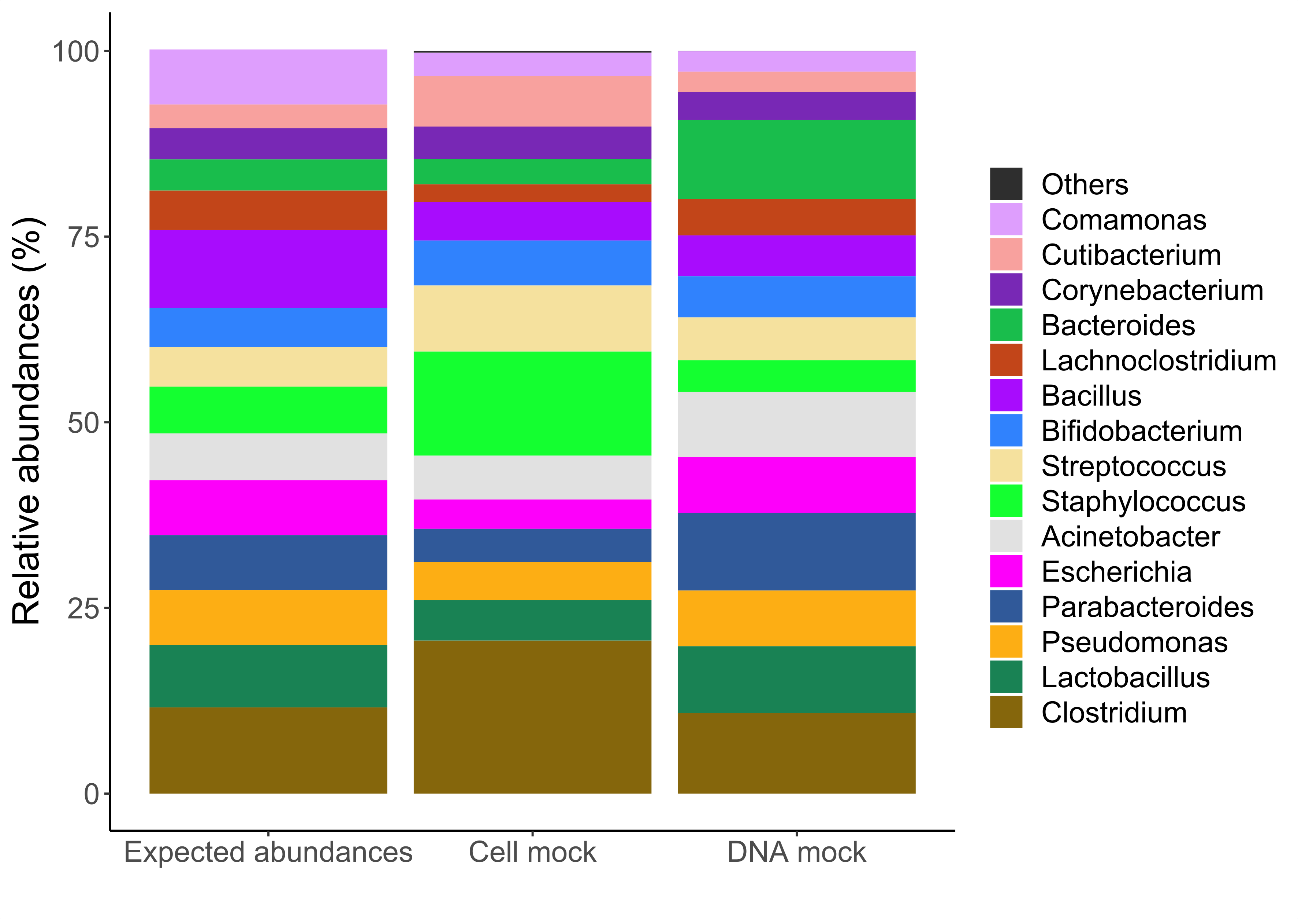
**

**Supplementary Fig. 6 Relative abundance of bacterial taxa present in the mock communities**

Each bar displays the relative abundance of the bacterial genus. The colours indicate the bacterial genus in the chart; bacteria not included in the mock are shown as "Others".


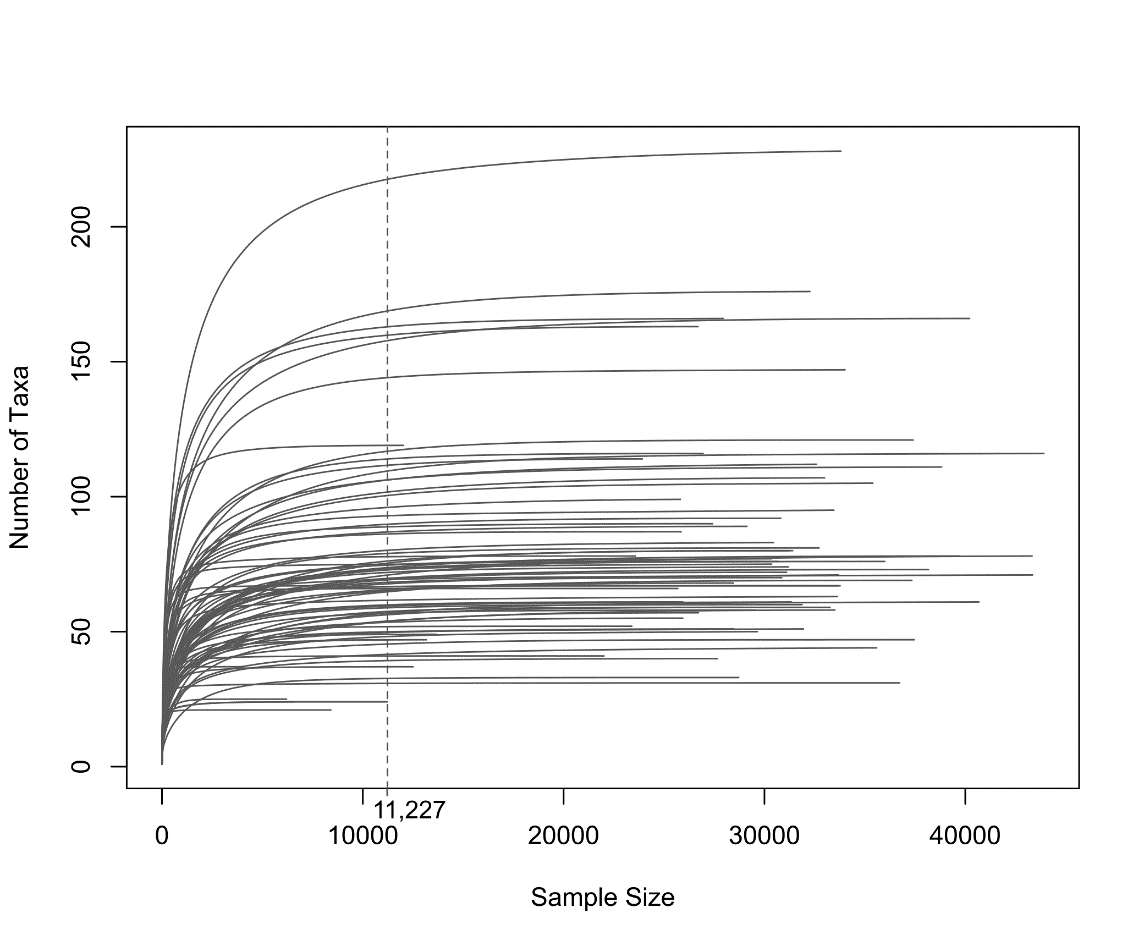


**Supplementary Fig. 7 Rarefaction curves of towel microbiomes.**

Rarefaction curves showing the richness of microbial communities in used towels. The dashed line indicates the number of reads (11,227), which was the smallest number of reads samples with more than 10,000 reads.
